# News and Notes

**Published:** 1905-08

**Authors:** 


### *NEWS AND NOTES.*

The Convocation of Oxford University proposes to confer on Dr. William Osler the degree of Doctor of Medicine.

A number of new cases of yellow fever have been reported from Panama, Colon, and intermediate points in the canal zone.

Dr. John B. Murphy has resigned the chair of surgery in the Northwestern University, Chicago, and will accept the same position at Rush Medical College.

The eighth annual meeting of the Association of Medical Librarians was held in Boston on Saturday, June 10, under the presidency of Dr. Jas. R. Chadwick, of Boston.

The Hanbury gold medal of the Pharmaceutical Society, London, has this year been awarded to Prof. Ernst Schmidt, professor of pharmaceutic chemistry in the University of Marburg.

The State University of Iowa has established a lectureship on tuberculosis in connection with the medical department of that institution, and has appointed Dr. J. W. Kime, of Fort Dodge, as lecturer.

Says an exchange: "The practical certainty that more than 1,000,000 immigrants will come into this country during the fiscal year ending with June, thus breaking all records, and the revelations made by agents of the Immigration Bureau in Europe concerning the methods employed by foreign governments to promote emigration of the criminal and unfit, have decided the President to urge Congress to make further restrictions. A heavy proportion of the newcomers are not desirable immigrants for political, economic, and moral reasons. One of the amendments that will probably be recommended is the raising of the head tax on immigrants to $25. This would bar thousands who are now admitted."

---

An exchange states that a bill has been introduced in the Iowa legislature forbidding "healers" to practice in the State under penalty of imprisonment in the penitentiary. The introducer of the bill has promiscd to withdraw it if the Eddyites will cure the doorkeeper of the House, who is afflicted with deafness. Some of the healers are not willing to accept the challenge, but others believe that this is the appointed time to make a cure and demonstrate their powers in the most public way, and propose to organize a concert of prayer and hard thinking for removal of the doorkeeper's belief that he can not hear.

---

The Japanese government, on the recommendation of the Mikado, has presented to Dr. Jokichi Takamine, formerly of the Tokio University, but now residing in New York, three handsome buildings, valued at $50,000, which were sent to America for the World's Fair at St. Louis. The gift, it is announced, is made in recognition not only of Dr. Takamine's services to the Imperial Japanese Commission at the Exposition, but also of important scientific and medical discoveries which have been applied during the present war with great benefit in the medical department of the Japanese army. We learn from the *Boston Medical and Surgical Journal* that Dr. Takamine will have the buildings set up at his summer house in Sullivan county, near Monticello, which he proposes to convert into a typical Japanese country-place. One of them, which is constructed of the choicest woods of Japan, was the scene of a luncheon served to the Emperor and Empress of Japan at the Osaka Exposition two years ago, and recently of receptions to President Roosevelt and to Prince Fushima when they were at St. Louis.

